# Supplementary material for: Neural correlates of the deployment of spatial attention, and their modulation by repetitive movements
Source: PLoS One. 2025 Oct 3;20(10):e0332583. doi: 10.1371/journal.pone.0332583 (PMC12494278; doi:10.1371/journal.pone.0332583)
Supplement: Attachment — Submitted filename: responsetoreviewers.docx [file pone.0332583.s001.docx]

Reviewer #1:

R1.1 The study relies on a binary self-report to classify participants as typically stimming or not, without using a validated scale or further behavioural detail. Authors should acknowledge this limitation explicitly in the Methods and Discussion (how this approach may mask important variability in frequency..). In addition, they have to cite and briefly describe at least one validated tool that could be used in future research to quantify stimming. Clarify in the Methods (p. 4–5) that the classification was based on a single questionnaire item and explain why this approach was chosen.

*We have now added:*

*“15/22 participants further reported they typically engaged in stimming behaviours, while 7 reported that they did not - this information was collected through a single yes/no question item asking if participants typically stim, due to a lack of available self-report stimming scales”*

*And:*

*“Another limitation of our investigation was our use of a binary self-report relating to whether participants typically engaged in stimming or not. If one were available, it would be beneficial to use a self-report scale designed to measure an individual’s stimming behaviours. However, at present, the available scales are not appropriate for this use. The two scales we identified were the Stereotyped Behaviour Scale (SBS; Rojahn et al., 2000) and a sub-section of the Aberrant Behaviour Checklist (ABC; Aman and Singh, 1986) - both scales require an observer to rate an individual’s behaviours, rather than being a self-report scale, making them unsuitable for use in this context. Further, the ABC has been criticized for subjective and unacceptable items - for example using condescending language such as ‘tantrums’ or describing individuals as ‘whiny’ (Chan and Baker, 2020). To facilitate further investigations into stimming it would be beneficial to develop an appropriate self-report scale, as those currently available are not suited for this purpose.”*

R1.2 The study compares individuals who typically stim to those who do not, yet the experimental stimming task was externally imposed and not fully naturalistic. Authors should expand on the potential difference between internally motivated (self-directed) vs externally instructed stimming. Consider whether this difference may explain the absence of beneficial effects on attention. As well as authors should suggest a future study design allowing naturalistic stimming and cite any precedent for such methods if available.

*We have now added:*

*“Considering that stimming is believed to address certain internal needs, stimming when externally prompted (i.e., not in direct response to these needs) may not generate an effect, because the individual doesn’t feel the need to self-regulate through stimming.”*

R1.3 Furthermore, the longer reaction times in the stimming condition may be confounded by the physical act of movement interfering with manual responses, rather than reflecting cognitive distraction. I recommend that the author should in the Discussion section be more explicit about this possible confound and its implications and propose a future adaptation of the task that avoids motor overlap.

*This is a good point. We have added the following section to the Discussion:*

*“Participants had to use one hand to stim while simultaneously responding to stimuli presented on the screen with the other hand - the incompatibility of the movements being performed simultaneously may therefore have caused, or contributed to, the increase in reaction times recorded. Future research into this phenomenon should modify the task to ensure stimming behaviours are compatible. Klier and Harris (1977), for example, used a learning task where children learnt behaviours either compatible or incompatible with stimming and found stimming itself did not have a detrimental effect on the ability to learn. A replication of this study could use a task in which participants respond verbally, for example, to limit the effect of incompatible movements.”*

R1.4 The analysis in Experiment 2 includes only 8 participants who do not typically stim, limiting the reliability of group comparisons. Author should acknowledge the limited power of subgroup comparisons and the potential for Type II errors and suggest conducting a power analysis to estimate the required sample size for detecting group differences in decoding accuracy or reaction time under this paradigm.
 *We have added:*

*“Overall, our relatively small sample size means that statistical power was low for these between group comparisons (increasing the risk of Type 2 errors), and we suggest that follow up studies should conduct power analyses to ensure they are better able to detect significant effects should they exist.”*

R1.5 Finally, while the study is well-cited, more recent citation and more clarity is needed in linking the behavioural neuroscience to the broader neurodiversity framework.

*We have added several additional citations to recent work in this field, both to the Introduction and Discussion sections. We also extend a section of the Discussion to cover links to the neurodiversity movement, which now reads:*

*“Particularly in the case of stimming, autistic adults protest against the elimination of these behaviours [6], and they have been posited as a significant feature of autistic culture and identity (Felepchuk, 2021; Petty and Ellis, 2024; Morris et al., 2025). There is also a growing shift in approaches to teaching autistic children, with new encouragement of accommodating and integrating stimming into the classroom environment (e.g., Tancredi and Abrahamson, 2024). Even though we have been unable to demonstrate in a controlled setting that these behaviours have benefits, it is important to listen to the community we aim to help and respect their wishes. This is particularly relevant in the context of the growing movement for acceptance of neurodiversity, and the push for inclusion of autistic perspectives in research, as stimming is a sensitive topic given historical attitudes and current social stigma surrounding it.”*
